# Alfalfa and Linseed Oil in Diet for Fattening Rabbits: Performance, Meat Quality, and Nutritional Characteristics

**DOI:** 10.3390/ani15233409

**Published:** 2025-11-26

**Authors:** Alessandro Dal Bosco, Cesare Castellini, Simone Mancini, Luigia Bosa, Francesca Di Federico, Lorenzo Nompleggio, Simona Huja, Simona Mattioli

**Affiliations:** 1 Department of Agricultural, Environmental and Food Science, University of Perugia, Borgo XX Giugno 74, 06125 Perugia, Italy; alessandro.dalbosco@unipg.it (A.D.B.); cesare.castellini@unipg.it (C.C.); lorenzo.nompleggio@studenti.unipg.it (L.N.); simona.huja@studenti.unipg.it (S.H.); 2 Department of Veterinary Sciences, University of Pisa, Viale delle Piagge 2, 56124 Pisa, Italy

**Keywords:** rabbit, dehydrated alfalfa, linseed oil, performance, meat quality

## Abstract

The research aimed to evaluate the effectiveness of adding linseed oil to an alfalfa-based diet for fattening rabbits. At 30 days, 360 hybrid rabbits were divided into three homogeneous groups and fed: Control feed composed of 15% wheat, 17% alfalfa hay, and 8% dehydrated alfalfa; experimental feed composed of 40% alfalfa hay and 30% dehydrated alfalfa (Alfalfa); another experimental feed composed of 38% alfalfa hay, 30% dehydrated alfalfa, and 2% linseed oil (Alfalfa+Linseed). The alfalfa-based diet showed higher crude fiber, but lower lipid, protein, and digestible energy content. Regarding performance, a reduction in slaughter weight was observed only in the Alfalfa group, while the addition of linseed oil eliminated the gap with the Control group. The lipid content of the meat was significantly lower in the Alfalfa group. Significant changes were observed in the fatty acid composition, with higher levels of n-3 precursors and derivatives of the n-3 series in the Alfalfa+Linseed group, followed by Alfalfa and Control. Furthermore, the addition of linseed oil worsened lipid oxidative stability. In conclusion, the proposed dietary treatment represents a promising strategy for producing rabbit meat with a high nutritional profile, without compromising productive performance.

## Simple Summary

The rabbit production sector is currently facing significant challenges related to sustainability, price volatility, competition between feed and food, and environmental impacts. This manuscript aims to evaluate the effectiveness of enriching an alfalfa-based diet for rabbit with linseed oil. The reason behind this research deals with the sustainability concept regarding feed ingredients as in recent decades there has been a strong reduction in the use of forage in rabbit diets. Indeed, this scenario does not match the sustainability issues for the rabbit, which being an herbivore, can digest fibers not competing with human nutrition (alfalfa). Therefore, diets high in forage should be recommended to reduce the dependence of the rabbit diet on grains and protein crops. Furthermore, to increase rabbit performance, an adjustment in fat-content of the diet has been studied (linseed oil addition).

## 1. Introduction

Rabbit production represents an interesting livestock opportunity for both Western and emerging countries [1,2]; however, some criticisms emerged concerning the image of the product and the production system. Rabbit is often considered as pet in some countries, moreover in intensive farms small cages are commonly used, as well as the high production cost compared to chicken meat and the difficulty of cooking its meat, determined a reduction of the European rabbit meat production by 37% over the past five years (2018–2022) across nearly all EU countries [3]. A recent Italian survey [3] indicated that the core fundamentals of the rabbit meat sector do not suggest any reversal in the declining demand, as it remains a niche market primarily targeted at mature consumers. Communication and promotion could be key drivers in revitalizing interest in this product. The report emphasizes that initiatives should be implemented to promote culinary education and raise consumer awareness about the nutritional benefits of rabbit meat and its alignment with sustainability goals. Consequently, it is essential to reform the rabbit farming system by gradually phasing out cages (e.g., replacing them with open parks) and adopting more animal-friendly and sustainable practices to improve the sector’s image and attract younger consumers [2].

One of the most important factors influencing the sustainability of rabbit production is the diet formulation [4]. Currently, the rabbit sector is highly dependent on conventional feed ingredients (cereals and soybean) [5], which contribute to price volatility, feed-food competition, and environmental impact. In recent decades, despite rabbits being strict herbivores, there has been a marked reduction in the use of forage in diets in order to improve performance and reduce feeding costs. This shift has also led to an increased reliance on industrial by-products, which are appealing from a circular economic perspective but may negatively affect rabbit health, the nutritional quality of the feed, and consequently, the characteristics of the meat.

A previous experiment [6] revealed improvements in the nutritional characteristics of the meat but, at the same time, a decline in growth performance, which could be partly attributed to the lower digestible energy content of the alfalfa diet. Accordingly, increasing the energy content of the diet through the addition of linseed oil could help reduce the performance gap and further enhance the nutritional quality of rabbit meat by boosting its n-3 fatty acid content [7,8].

Numerous studies have investigated the use of linseed in animal nutrition. Recently, the results of an international project called ΩRABBIT, which examined the effects of dietary supplementation with flax and algae, led to the establishment of a consortium dedicated to producing rabbit meat enriched with omega-3 (ΩRabbit meat [9,10,11]). Linseed contains high levels of α-linolenic acid (C18:3n-3, ALA), which is notably prone to oxidative degradation. If used under suboptimal conditions, such as excessive inclusion levels, poor ingredient quality, or insufficient antioxidant protection, it may negatively impact shelf life, nutritional value, color, sensory attributes, and, ultimately, consumer acceptance. For all these reasons, and in pursuit of a balance between sustainability and nutritional value, this study aimed to evaluate the effectiveness of enriching an alfalfa-based diet with linseed oil, focusing on productive performance, as well as nutritional and health-related traits.

## 2. Materials and Methods

### 2.1. Animals and Experimental Design

The trial was carried out at a commercial rabbitry (Brachino Patrizia, Bagnoregio, VT, Italy). Animal experimentation complied with the ARRIVE guidelines, and it was carried out in accordance with the Guiding Principles in the Use of Animals and approved by the Animal Ethics Monitoring Committee of the University of Perugia (Authorization n° 51/2024-PR; protocol n. 349569).

At weaning (30 days), three hundred sixty (360) hybrid rabbits (Martini Company), of both sexes (M:F, 1:1), were divided into three homogeneous groups and fed one of the following diets: control feed composed of 150 g/kg wheat, 170 g/kg alfalfa hay, and 80 g/kg dehydrated alfalfa (Control); experimental feed composed of 400 g/kg alfalfa hay and 300 g/kg dehydrated alfalfa (Alfalfa); and another experimental feed composed of 380 g/kg alfalfa hay, 300 g/kg dehydrated alfalfa, and 20 g/kg linseed oil (Alfalfa+Linseed) (Table 1).

In aiming to maximize the use of alfalfa, it was not possible to maintain nutritional equivalence among the diets in terms of crude protein, digestible energy, and fiber fractions. All diets were formulated in accordance with the dietary recommendations for fattening rabbits [13]. The rabbits were kept in a closed shelter with automated systems for controlling temperature and ventilation (18–25 °C; 0.5–1.0 m/s) and reared in 180 bicellular wire cages (28 × 40 × 30 cm height; 60 replications per group).

The feed intake was recorded daily in every cage, and live weight was recorded weekly to determine the growth rate and the feed efficiency (calculated as the ratio between the daily intake and the daily weight-feed to gain ratio). The health status of rabbits and mortality were monitored and registered daily.

At 80 days of age, all rabbits (*n* = 346; see mortality data) were slaughtered in an EU-approved facility, 12 h after feed withdrawal, following the EU Regulation 1099/2009. The animals were sacrificed by severing the carotid arteries and jugular veins following electro-stunning, and twenty carcasses per group were selected and processed according the procedures recommended by the World Rabbit Science Association (WRSA), as described by Blasco, Mateu, Ouhayoun, and Masoero [14]. The carcass sizes and weights were recorded along with the weights of interscapular and perivisceral fat, liver, and kidneys.

The *longissimus thoracis et lumborum* (LTL) muscles (located between the 1st and 7th lumbar vertebrae) were excised from both sides of the refrigerated carcasses (stored for 24 h at 4 °C) and trimmed of all external fat and epimysial connective tissue. The two muscles were then stored at −80 °C in the Laboratory of the Department of Agricultural, Food, and Environmental Sciences (University of Perugia) for subsequent analyses of chemical composition, fatty acid profile, and oxidative status, which were performed about 2 weeks later.

### 2.2. Chemical Analyses

#### 2.2.1. Proximate Composition of Diet and LTL Meat

The chemical composition of the feed (including moisture, protein, lipid, and ash contents) was determined according to the methods of the Association of Official Analytical Chemists (AOAC) [15] and expressed as the mean of three replicates. Crude fiber, neutral detergent fiber (NDF), acid detergent fiber (ADF), and acid detergent lignin (ADL) were analyzed following the procedures described by Van Soest et al. [16].

The proximate composition of the meat—including moisture, protein, lipid, and ash contents—was determined according to AOAC methods [15]. Briefly, the moisture content was determined by oven-drying of the meat samples (125 °C for 24 h) (method 950.46). Fat content was determined gravimetrically using an ether solvent extraction (method 960.30). Total lipids were extracted in duplicate from 5 g of each homogenized sample and quantified gravimetrically [17]. Protein content was obtained by multiplying the total Kjeldahl nitrogen (method 992.15) by a conversion factor of 6.25. Ash content was measured using a muffle furnace at 600 °C, following AOAC method 923.03.

#### 2.2.2. Fatty Acid Composition of Diet and LTL Meat

The fatty acid composition of feed and LTL meat was determined by gas chromatography. Total lipids were extracted from 10 g of each homogenized sample, following the method of Folch et al. [17] and subsequently esterified according to Christie [18]. One mL of each solution containing Fatty Acid Methyl Ester (FAME) was transferred into vials for gas chromatographic analysis. Separation of FAMEs was performed using a Varian gas chromatograph (CP-3800) equipped with a flame ionization detector (FID) and an Agilent capillary column (100 m × 0.25 mm, CPS Analitica, Milan, Italy) coated with a DB-Wax stationary phase (film thickness of 0.25 µm). Injector and detector temperatures were set at 270 °C and 300 °C, respectively. Helium was used as the carrier gas at a flow rate of 0.6 mL/min. The oven temperature was programmed as follows: from 40 °C (1 min hold) to 163 °C (10 min hold) at a ramp of 2 °C/min; then to 180 °C (7 min hold) at 1.5 °C/min; to 187 °C (2 min hold) at 2 °C/min; and finally to 230 °C (25 min hold) at 3 °C/min. Individual FAMEs were identified by comparing their retention times with those of a commercially available standard mixture (FAME mix Supelco 2560, Sigma-Aldrich, Taufkirchen, Germany). C21:0 methyl ester (CAS No. 2363-71-5; Merck H5149, Darmstadt, Germany), eluted under the same conditions as the samples, was used as an internal standard (1 mg/100 µL of added solution).

Peak areas were used to calculate the proportion of each fatty acid, with qualitative data expressed as percentages of total fatty acids. For quantitative analysis (mg/100 g of meat), the method described by Vahmani et al. [19] was applied. The average amount of each fatty acid was used to calculate the total content of saturated (SFA), monounsaturated (MUFA), polyunsaturated (PUFA), and long-chain polyunsaturated fatty acids (LCP; C > 20).

To facilitate a more accurate interpretation of data trends, Δ values between the absolute fatty acid contents (mg/100 g of meat) of the Control and Alfalfa groups were calculated.

#### 2.2.3. Estimated Lipid Index

Based on our previous study on fatty acid indexing [20], a Healthy Fatty Index (HFI) was calculated, starting from the fatty acid quantities (mg/100 of meat) as follows (1):

HFI = ((∑MUFA × 2) + (∑n-6 PUFA × 4) + (∑n-3 PUFA × 8) + (∑n-3 PUFA/∑n-6 PUFA))/(∑SFA + (∑MUFA × 0.5) + (∑n-6 PUFA × 0.25) + (∑n-3 PUFA × 0.125) + (∑n-6 PUFA/∑n-3 PUFA))(1)

The index was partially based on the equations proposed by Ulbricht and Southgate [21] but was modified to reflect updated nutritional assumptions [22].

#### 2.2.4. Lipid Oxidation of LTL Meat

Lipid oxidation was assessed using a spectrophotometer set at 532 nm (Shimadzu Corporation UV-2550, Kyoto, Japan), which measured the absorbance of thiobarbituric acid reactive substances (TBARS) against a 1,1,3,3-tetraethoxypropane calibration curve [23]. Oxidation products were quantified and expressed as malondialdehyde equivalents (µg MDA/g).

### 2.3. Statistical Analyses

For the statistical analysis of growth performance, a mixed model was used, with diet as a fixed effect and cage as a random effect (STATA, 2015; PROC MIXED [24]). Carcass traits and qualitative meat characteristics were analyzed using a one-way model, with diet as the fixed effect. Differences in mortality rate were evaluated using the χ^2^ test.

Multiple comparisons were performed using post hoc Bonferroni’s range test, with significance levels set at *p* < 0.05 and *p* < 0.001.

## 3. Results

The different feed formulations resulted in distinct chemical characteristics. In particular, the alfalfa-based diets exhibited higher crude fiber content and elevated levels of all fiber fractions (NDF, ADF, and ADL), along with lower lipid, protein, and digestible energy values. These differences were less pronounced in the Alfalfa+Linseed diet (Table 1).

The fatty acid composition of the diets, expressed in mg of fatty acids per 100 g of feed, was influenced by the lipid content. The addition of linseed oil resulted in lipid levels comparable to the Control diet, while the Alfalfa diet showed significantly lower lipid content (3.26 and 2.98 mg/100 g in Control and Alfalfa+Linseed, respectively, vs. 1.80 mg/100 g in Alfalfa). Despite these similarities in total lipid content, the three experimental diets differed in fatty acid profiles, particularly with respect to ALA and linoleic acid (C18:2n-6, LA).

Regarding productive performance (Table 2), a significant reduction (*p* < 0.001) in slaughter weight was observed only in the Alfalfa group. However, the addition of linseed oil to the alfalfa-based diet eliminated this gap, achieving results comparable to the Control group. Similar trends were observed for feed conversion ratio and carcass weight. Notably, post-weaning mortality showed a progressive reduction: It was zero in the Alfalfa+Linseed group, slightly higher in the Alfalfa group (3.33%), and reached typical levels in the Control group (8.34%). No significant differences were found in carcass yield among the groups.

In Table 3, the proximate composition of LTL meat is reported. The lipid content of the LTL muscle was significantly lower in the Alfalfa group compared to both the Control and Alfalfa+Linseed groups (*p* < 0.05). No significant differences were observed in the other chemical components of the meat.

The fatty acid composition is reported in Table 4. Significant changes in fatty acid composition (% of total fatty acids) were observed, with higher levels of the n-3 precursor α-linolenic acid and its derivatives, eicosapentaenoic acid (C20:5n-3, EPA), docosapentaenoic acid (C22:5n-3, DPA), and docosahexaenoic acid (C22:6n-3, DHA), in the Alfalfa+Linseed group, followed by the Alfalfa and Control groups (*p* < 0.001). Regarding the n-6 series, LA and arachidonic acid (C20:4n-6, AA,) were significantly higher in the Control group compared to the other two dietary treatments.

When the fatty acids were expressed in mg/100 g of muscle (Table 5), the differences appeared greater due to the contemporary effect of the lipid content and the fatty acid profile of diets. Alfalfa+Linseed group showed higher levels of ALA, EPA, DHA, and total n-3 fatty acids. As a result, the n-6/n-3 ratio progressively decreased (23.97, 4.59 and 2.40) in Control, Alfalfa, and Alfalfa+Linseed groups, respectively.

**Figure 1:**
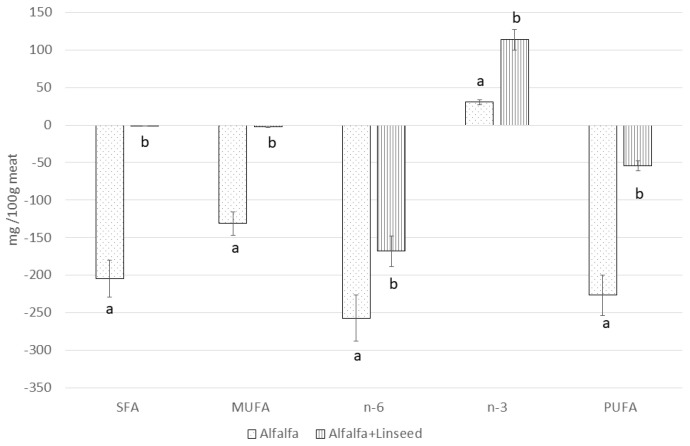
Variation (mg/100 g of meat) with respect to Control (equal to = 0) in the main fatty acid categories (SFA, MUFA, PUFA n-3, and n-6) of *longissimus thoracis et lumborum* muscle of rabbit fed different alfalfa-based diets. a, b: Values differ between groups for *p* < 0.05. SFA = saturated fatty acids, MUFA = monounsaturated fatty acids, PUFA = polyunsaturated fatty acids, n-6 = polyunsaturated fatty acids from n-6 series, and n-3 = polyunsaturated fatty acids from n-3 series.

**Figure 2:**
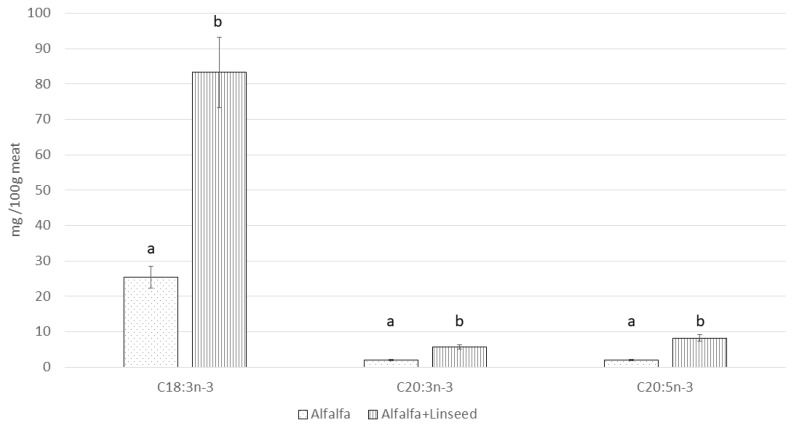
Variation (mg/100 g meat) with respect to Control (equal to = 0) in n-3 fatty acids in *longissimus thoracis et lumborum* muscle of rabbit fed different alfalfa-based diets. a, b: Values differ between groups for *p* < 0.05.

To facilitate a more accurate interpretation of data trends, the Δ values between the absolute fatty acid contents (mg/100 g of meat) of the Control and the two Alfalfa groups were calculated (Figure 1 and Figure 2). In Figure 1, it can be observed that the SFA and MUFA contents of the Alfalfa+Linseed group are similar to Control, while PUFA and n-6 showed a certain reduction; on the contrary, n-3 levels were significantly higher. As such, the Δ values against the Alfalfa group, by virtue of the very different lipid contents, proved to be much higher for all the classes analyzed. The profile of n-3 PUFA in the meat drastically changed in the Alfalfa groups, with greater amounts of ALA, EPA, and DHA, mainly when linseed was added to the diet (Figure 2).

**Figure 3:**
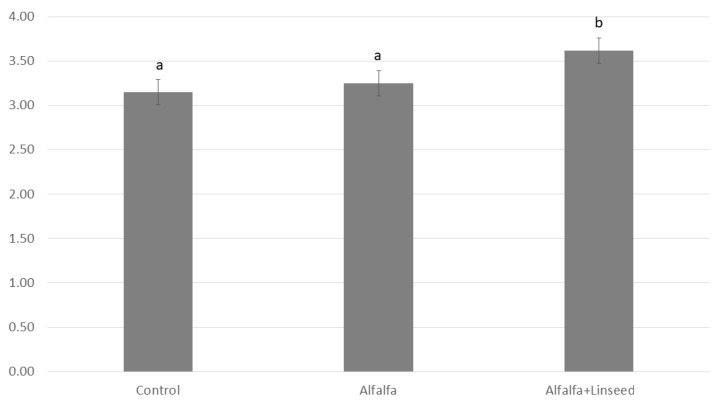
Healthy Fatty index (HFI) of *longissimus thoracis et lumborum* muscle obtained from rabbits fed different experimental diets. Mean ± SEM is reported. a, b: Values differ between groups for *p* < 0.05.

Regarding HFI (Figure 3), increasing values were observed in both alfalfa-fed groups, with the highest value recorded in the Alfalfa+Linseed group.

The addition of linseed enhanced the LCPn-3/precursor ratio in rabbit meat, indicating greater efficiency of the Alfalfa-based diets in enriching meat with long-chain n-3 polyunsaturated fatty acids (Table 6). Conversely, both Alfalfa-enriched diets reduced the LCPn-6/precursor ratio.

**Figure 4:**
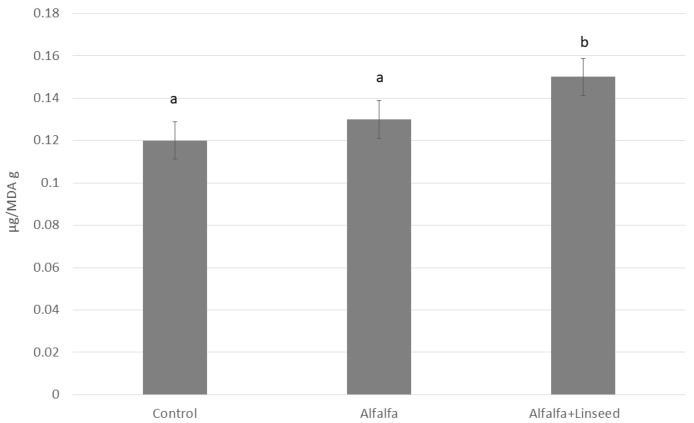
Oxidative status (µg MDA/g) of *longissimus thoracis et lumborum* muscle of rabbits fed different experimental diets. Mean ± SEM is reported. a, b: Values differ between groups for *p* < 0.05.

Lipid stability (Figure 4) was influenced by the inclusion of linseed oil. While the stability of meat from the Control and Alfalfa groups did not differ significantly, the Alfalfa+Linseed group exhibited increased lipid oxidation.

## 4. Discussion

As already outlined, the objective of this study was not to dissect the effects of individual dietary ingredients in isolation, but rather to propose an alternative feeding strategy that prioritizes the use of components non-competitive with human nutrition. A natural limitation of this approach is that it does not permit a detailed evaluation of the specific roles and interactions among nutritional factors (e.g., fiber, protein, and energy) in shaping the observed traits.

In the present investigation, to overcome the performance decline observed in our previous research [6,25,26] while maintaining the beneficial effects on rabbit health, 2% flaxseed oil was added to the alfalfa-based diet. This modification improved digestible energy (2180 and 2341 kcal/kg in Alfalfa and Alfalfa+Linseed, respectively) and resulted in comparable slaughter weights, reduced mortality, and feed conversion efficiency similar to the control group. Notably, the mortality rate was very low in both the experimental groups. Previous analysis of the post-weaning microbiota composition revealed a progressive improvement in microbial profiles among animals receiving alfalfa-based diets [25]. This enhanced microbiota composition, characterized by an increase in Firmicutes and a decrease in *Bacteroides* and *Proteobacteria*, may help explain the lower mortality rates observed in the Alfalfa groups. Thus, the emergence of distinct microbial differences between groups fed different diets, particularly when linked to high-fiber intake, can be interpreted as a positive indicator of improved gut microbiota stability and functionality, all important factors for the long-term health of the animal, as also highlighted by Combes et al. [27,28].

Moreover, the lipid content of the rabbit meat in the Alfalfa+Linseed group was comparable to the Control group and higher than in the Alfalfa group, demonstrating that 2% linseed oil is sufficient to compensate for the energy deficit of the alfalfa diet. The leaner meat of rabbits fed the alfalfa diet was likely attributable to the high proportion of alfalfa, which reduced the digestible energy of the diet. Although this led to increased consumption of pelleted feed, it was insufficient to offset the energy deficit. Dal Bosco et al. [6] also noted that the alfalfa-based diet was considerably richer in crude fiber and its fractions, while being low in starch and energy substrates [28,29].

Regarding the fatty acid profile (% of total fatty acids), higher levels of the n-3 precursor α-linolenic acid (ALA) and its derivatives (eicosapentaenoic acid—EPA, docosapentaenoic acid—DPA, and docosahexaenoic acid—DHA) were found in the Alfalfa+Linseed group, followed by the Alfalfa and Control groups (*p* < 0.001). Capra et al. [30], using a comparable dietary strategy, reported similar findings, particularly in ALA content (1.6% vs. 3.1%, respectively) in the intramuscular fat of rabbits fed control or alfalfa-based diets.

This trend also influenced the fatty acid of meat (mg/100 g of meat) and the resulting Healthiness Fatty Index (HFI), with higher values observed in both alfalfa-fed groups and the highest recorded in the Alfalfa+Linseed. This confirms the superior nutritional quality of alfalfa-enriched meat compared to standard meat. Indeed, the HFI index effectively differentiates fat quality based on its n-3 and n-6 fatty acid composition, even when differences in fatty acid are relatively small [20,22]. Rabbit meat is already considered a healthy product due to its higher PUFA content and lower lipid content compared to other animal products [31]. A careful categorization of fatty acid classes based on lipid content, as achieved through HFI, allows differentiation of similar products, such as meat from control and alfalfa-based diets. To achieve optimal nutritional outcomes, it is not sufficient to produce meat with an excellent fatty acid profile alone; maintaining a minimum threshold of fat content is necessary to ensure adequate intake of beneficial fatty acids. Furthermore, flaxseed addition enhanced the LCPn-3/precursor ratio, indicating greater efficiency of alfalfa-based diets in enriching meat with n-3 long-chain polyunsaturated fatty acids (LCP).

Lipid stability (Figure 4) was also influenced by the inclusion of linseed oil. While the stability of meat from the Control and Alfalfa groups did not differ significantly, the Alfalfa+Linseed group exhibited increased lipid oxidation. This outcome is expected, given that lipids are the primary substrates for oxidation by reactive oxygen species (ROS) [32], and fatty acids with multiple unsaturation bonds, such as PUFA, are more susceptible to ROS-induced oxidation [33]. Linseed provided an increased amount of PUFA, mainly ALA, which enriched the n-3 PUFA content of meat. To preserve the shelf life of rabbit meat, especially when pro-oxidant compounds (i.e., linseed) are included in the diet, further in vivo supplementation with antioxidant compounds is recommended, such as at least 200 mg/kg of feed of tocopheryl acetate [33,34,35], or natural additives including spices [36], goji berries [37], and various agro-industrial by-products [36,38,39,40]. In this study, a standard addition of vitamin-like ingredients was applied; however, targeted antioxidant supplementation may be advisable.

Notably, the availability of substantial ALA also led to an increase in LCPn-3. This is supported by the higher LCPn-3/precursor ratio, which underscores the rabbit’s capacity to elongate and desaturate ALA when sufficiently supplied [41,42,43]. Fatty acids from the n-3 series are considered bioactive compounds that help reduce in vivo inflammation, thereby improving rabbit health. Furthermore, the literature reports that linseed may enhance the flavor, texture, and nutritional/antioxidant properties of various meat-based products when administered at concentrations above 5% [44]. Accordingly, further investigations are in progress to evaluate the effect of linseed addition on the sensory characteristics of rabbit meat, since our previous study [6] demonstrated no differences in consumer perception of meat from rabbits fed a simple alfalfa-based diet.

## 5. Conclusions

This dietary approach represents a promising and non-competitive strategy with respect to human nutrition, enabling the production of rabbit meat with a high nutritional profile without compromising animal performance. Such a trade-off underscores the importance of evaluating feeding strategies not only in terms of productivity but also in relation to animal welfare and long-term sustainability. The use of non-competitive feed resources, such as alfalfa, aligns with broader One Health and One Welfare frameworks, as it reduces competition with human food chains while supporting greater resilience in rabbit production systems.

The findings presented here provide a novel contribution, demonstrating the synergistic role of alfalfa and linseed oil in enhancing both the nutritional and energetic quality of rabbit feed. Notably, this combination improves the energy density of the diet while significantly enriching the meat with health-promoting fatty acids, particularly α-linolenic acid (ALA). The availability of ALA from these two ingredients plays a pivotal role in elevating the nutritional value of rabbit meat, highlighting their complementarity in sustainable feeding practices. Nevertheless, adequate antioxidant supplementation remains advisable to preserve meat quality and shelf life under conditions of increased PUFA enrichment.

## Figures and Tables

**Table 1:** Formulation, chemical composition, and fatty acid profile of experimental diets.

Diets	Control	Alfalfa	Alfalfa+Linseed
Ingredients (g/kg)			
Dehydrated alfalfa meal	80.00	400.00	380.00
Alfalfa hay	170.00	300.00	300.00
Wheat meal	150.00	140.00	140.00
Wheat bran	120.00	-	-
OGM soybean meal	140.00	-	-
Sunflower meal	80.00	83.00	83.00
Dried beet pulp	20.00	35.50	35.00
Barley meal	210.00	25.50	25.00
Linseed oil ^1^			20.00
Soybean oil	10.00	-	-
Mineral-vitamin premix ^2^	10.00	10.00	10.00
Limestone	5.00	5.00	5.00
Marine salt	5.00	2.00	2.00
Chemical composition (g/100 g D.M.)			
Crude protein (CP)	16.40	15.80	15.60
Crude fiber	15.60	22.00	21.40
Ether extract (EE)	3.26	1.80	2.98
Ash	7.09	7.95	7.90
NDF	30.30	40.40	38.40
ADF	18.60	25.40	23.20
Hemicellulose	11.70	15.00	13.20
Cellulose	14.00	23.50	18.30
ADL	1.88	4.57	4.23
Digestible energy (DE) (kcal/kg) ^3^	2450	2180	2341
Fatty acids (mg/100 g diet)			
SFA	587.56	246.62	487.25
MUFA	738.81	193.89	687.23
PUFA	1401.30	765.96	1504.21
C18:2n-6	1274.94	561.19	1054.23
C18:3n-3	83.23	169.32	268.36
Total n-6	1318.08	596.64	1254.02
Total n-3	83.23	178.15	286.22

^1^ Bleu and Marine, Bretagne, France, 100% linseed oil: containing 60% n-3, 27% n-6, and 13% n-9 PUFA. ^2^ Added per kg: vit. A U.I. 11.000; vit. D_3_ U.I. 2.000; vit. B_1_ 2.5 mg; vit. B_2_ 4 mg; vit. B_6_ 1.25 mg; vit. B_12_ 0.01 mg; vit. E 25 mg; biotine 0.06 mg; vit. K 2.5 mg; niacine 15 mg; folic ac. 0.30 mg; d-pantotenic ac. 10 mg; coline 600 mg; Mn 60 mg; Cu 3 mg; Fe 50 mg; Zn 15 mg; I 0.5 mg; Co 0.5 mg; lysine 50 mg; methionine 40 mg^.^ NDF: Neutral Detergent Fiber. ADF: Acid Detergent Fiber. ADL: Acid Detergent Lignin. ^3^ DE: Estimated with the following equation: 14.2 − 0.205 ADF + 0.218 EE + 0.057 CP (Fernández-Carmona et al. [12]). SFA = saturated fatty acids; MUFA: monounsaturated fatty acids; PUFA: polyunsaturated fatty acids.

**Table 2:** Performance and carcass characteristics of rabbits fed different experimental diets.

	Control	Alfalfa	Alfalfa+Linseed	RMSE/X^2^ *	*p*
Weaning weight, 30 d (g) ^1^	618.3 ± 61.0	627.2 ± 60.7	620.3 ± 59.3	10.2	0.515
Slaughtering weight, 80 d (g) ^2^	2723.9 ^B^ ± 225.4	2484.2 ^A^ ± 255.1	2708.5 ^B^ ± 248.1	35.2	0.001
Feed intake, (g/d)	137.50 ^A^ ± 12.83	151.97 ^B^ ± 14.17	146.17 ^AB^ ±14.10	9.24	0.001
Feed conversion ratio	3.20 ^a^ ± 0.30	4.01 ^b^ ± 0.38	3.43 ^a^ ± 0.31	0.84	0.004
Mortality (%)	8.34 ^c^	3.33 ^b^	0.00 ^a^	2.41 *	0.002
Carcass yield (%) ^3^	56.79 ± 3.12	57.88 ± 3.87	57.67 ± 4.10	4.23	0.100
Carcass weight (g) ^3^	1574.8 ^b^ ± 155.1	1429.5 ^a^ ± 138.8	1562.5 ^b^ ± 142.2	16.4	0.025

^1^ *n* = 120/group; ^2^ *n* = 110 Control, 116 Alfalfa, 120 Alfalfa+Linseed; ^3^ *n* = 20/group; a, b, c: on the same row, values differ for *p* < 0.05. A, B: On the same row, values differ for *p* < 0.001. Values are reported as mean ± SEM (standard error of the mean). RMSE: root mean square error. *: X^2^.

**Table 3:** Chemical composition (g/100 g meat) of *longissimus thoracis et lumborum* muscle of rabbit fed different experimental diets.

	Control	Alfalfa	Alfalfa+Linseed	RMSE	*p*
Moisture	74.12 ± 3.05	75.01 ± 2.55	74.31 ± 3.10	2.28	0.357
Protein	22.51 ± 1.08	22.43 ± 1.00	22.61 ± 1.85	1.02	0.107
Lipids	1.72 ^B^ ± 0.08	1.05 ^A^ ± 0.08	1.65 ^B^ ± 0.06	0.12	0.005
Ash	1.65 ± 0.10	1.51 ± 0.09	1.43 ± 0.08	0.18	0.847

*n* = 20 per group. A, B: On the same row, values differ for *p* < 0.05; values are reported as mean ± SEM (standard error of the mean). RMSE: root mean square error.

**Table 4:** Fatty acid composition (g/100 g of fatty acids) of *longissimus thoracis et lumborum* muscle of rabbit fed different experimental diets.

Fatty Acids	Control	Alfalfa	Alfalfa+Linseed	RMSE	*p*
C14:0	2.01 ± 0.46	2.04 ± 0.33	2.36 ± 0.26	0.18	0.253
C16:0	28.47 ± 1.61	28.85 ± 1.88	28.05 ± 2.04	1.54	0.961
C18:0	8.12 ^a^ ± 0.93	9.45 ^b^ ± 0.82	8.63 ^a^ ± 0.79	0.58	0.015
Others	1.29 ^a^ ± 0.15	2.01 ^b^ ± 0.18	2.45 ^b^ ± 0.21	0.12	0.028
Total SFA	39.82 ± 3.02	42.35 ± 3.24	41.49 ± 3.69	3.85	0.184
C14:1n-6	0.10 ± 0.02	0.10 ± 0.01	0.16 ± 0.03	0.01	0.662
C16:1n-7	1.98 ^A^ ± 0.18	2.31 ^A^ ± 0.22	3.22 ^B^ ± 0.28	0.31	0.001
C18:1n-9	22.35 ± 1.05	22.85 ± 2.41	21.47 ± 2.11	5.06	0.187
Others	0.43 ± 0.05	1.75 ± 0.20	0.95 ± 20.09	0.49	0.247
Total MUFA	24.76 ± 1.23	25.91 ± 2.03	25.64 ± 1.98	3.11	0.113
C18:2n-6	28.08 ^c^ ± 1.98	24.06 ^b^ ± 2.08	20.02 ^a^ ± 2.02	3.87	0.004
C20:2n-6	0.48 ± 0.03	0.25 ± 0.02	0.28 ± 0.03	0.12	0.457
C20:4n-6	5.48 ^b^ ± 0.49	2.47 ^a^ ± 0.19	3.03 ^a^ ± 0.29	1.16	0.008
Total n-6	34.04 ^B^ ± 2.21	26.78 ^A^ ± 1.82	23.33 ^A^ ± 2.01	3.02	<0.001
C18:3n-3	1.27 ^A^ ± 0.14	5.02 ^B^ ± 0.50	7.36 ^C^ ± 0.61	0.91	<0.001
C20:3n-3	0.03 ^A^ ± 0.00	0.25 ^B^ ± 0.02	0.43 ^C^ ± 0.03	0.42	<0.001
C20:5n-3	0.08 ^A^ ± 0.00	0.35 ^B^ ± 0.02	0.68 ^C^ ± 0.03	0.08	<0.001
C22:6n-3	0.07 ^A^ ± 0.00	0.22 ^B^ ± 0.02	1.27 ^C^ ± 0.12	0.04	<0.001
Total n-3	1.42 ^A^ ± 0.15	5.84 ^B^ ± 0.42	9.47 ^C^ ± 0.78	1.81	<0.001
Total PUFA	35.46 ^b^ ± 2.86	32.62 ^a^ ± 3.12	33.07 ^a^ ± 3.09	3.21	0.004

*n* = 20 per group. a, b, c: On the same row, values differ for *p* < 0.05. A, B, C: On the same row, values differ for *p* < 0.001. Values are reported as mean ± SEM (standard error of the mean). RMSE: root mean square error. SFA: saturated fatty acids; MUFA: monounsaturated fatty acids; PUFA: polyunsaturated fatty acids.

**Table 5:** Fatty acid composition (mg/100 g of meat) of *longissimus thoracis et lumborum* muscle of rabbit fed different experimental diets.

Fatty Acids	Control	Alfalfa	Alfalfa+Linseed	RMSE	*p*
C14:0	28.94 ^b^ ± 3.25	17.75 ^a^ ± 2.02	32.57 ^b^ ± 3.14	82.18	0.002
C16:0	409.97 ^b^ ± 28.21	251.00 ^a^ ± 24.55	387.09 ^b^ ± 30.01	51.18	0.009
C18:0	115.92 ^b^ ± 11.78	82.22 ^a^ ± 9.46	119.09 ^b^ ± 10.08	38.14	0.021
Others	18.58 ± 2.28	17.49 ± 1.52	33.81 ± 3.23	8.42	0.245
Total SFA	573.41 ^b^ ± 49.07	368.45 ^a^ ± 30.88	572.56 ^b^ ± 50.27	75.14	0.004
C14:1n-6	1.44 ^ab^ ± 0.12	0.87 ^a^ ± 0.07	2.21 ^b^ ± 0.23	0.48	0.002
C16:1n-7	28.51 ^B^ ± 2.42	20.10 ^A^ ± 1.82	44.44 ^C^ ± 3.77	8.15	0.001
C18:1n-9	321.84 ^B^ ± 30.91	190.10 ^A^ ± 20.02	296.29 ^B^ ± 27.71	78.19	0.001
Others	6.19 ^a^ ± 0.63	15.23 ^b^ ± 1.09	13.11 ^b^ ± 1.10	3.75	0.004
Total MUFA	356.54 ^b^ ± 30.17	225.42 ^a^ ± 23.64	353.83 ^b^ ± 29.51	90.36	0.008
C18:2n-6	404.35 ^C^ ± 35.52	209.32 ^A^ ± 26.24	276.28 ^B^ ± 30.06	85.19	<0.001
C20:2n-6	6.91 ^B^ ± 0.41	2.18 ^A^ ± 0.22	3.86 ^AB^ ± 0.46	1.74	<0.001
C20:4n-6	78.91 ^b^ ± 5.08	21.49 ^a^ ± 2.87	41.81 ^ab^ ± 3.64	9.12	0.002
Total n-6	490.18 ^C^ ± 50.03	232.99 ^A^ ± 30.56	321.95 ^B^ ± 30.99	97.25	<0.001
C18:3n-3	18.29 ^A^ ± 0.99	43.67 ^B^ ± 3.11	101.57 ^C^ ± 8.76	15.24	<0.001
C20:3n-3	0.29 ^A^ ± 0.02	2.18 ^B^ ± 0.17	5.93 ^C^ ± 0.53	1.36	<0.001
C20:5n-3	1.15 ^A^ ± 0.17	3.05 ^B^ ± 0.32	9.38 ^C^ ± 0.88	1.94	<0.001
C22:6n-3	0.72 ^A^ ± 0.06	1.91 ^B^ ± 0.24	17.53 ^C^ ± 1.24	3.21	<0.001
Total n-3	20.45 ^A^ ± 2.00	50.81 ^B^ ± 4.26	134.41 ^C^ ± 10.87	18.49	<0.001
Total PUFA	510.62 ^b^ ± 41.02	283.79 ^a^ ± 29.03	456.37 ^b^ ± 30.61	99.88	0.005
n-6/n-3	23.97 ^C^ ± 2.65	4.59 ^B^ ± 0.38	2.40 ^A^ ± 0.22	6.23	0.001

*n* = 20 per group. a, b: On the same row, values differ for *p* < 0.05. A, B, C: On the same row, values differ for *p* < 0.001. Values are reported as mean ± SEM (standard error of the mean). RMSE: root mean square error. SFA: saturated fatty acids; MUFA: monounsaturated fatty acids; PUFA: polyunsaturated fatty acids.

**Table 6:** Long-chain polyunsaturated fatty acids (LCP)/precursor ratio of *longissimus thoracis et lumborum* muscle of rabbit fed different experimental diets.

	Control	Alfalfa	Alfalfa+Linseed	RMSE	
PUFA n-3	11.81 ^a^ ± 0.98	16.35 ^b^ ± 1.43	32.33 ^c^ ± 2.12	1.58	0.023
PUFA n-6	21.22 ^c^ ± 1.41	11.32 ^a^ ± 0.87	16.50 ^b^ ± 01.36	1.20	0.042

*n* = 20 per group. a, b, c: On the same row, values differ for *p* < 0.05. Values are reported as mean ± SEM (standard error of the mean). RMSE: root mean square error.

## Data Availability

The data presented in this study are available on request from the corresponding author.
